# Separation of Traits and Extreme Response Style in IRTree Models: The Role of Mimicry Effects for the Meaningful Interpretation of Estimates

**DOI:** 10.1177/00131644231213319

**Published:** 2023-12-22

**Authors:** Viola Merhof, Caroline M. Böhm, Thorsten Meiser

**Affiliations:** 1 University of Mannheim, Germany; 2 Rhineland-Palatinate Technical University of Kaiserslautern-Landau, Germany

**Keywords:** IRTree models, response styles, multidimensional item responding, meaningful model parameters

## Abstract

Item response tree (IRTree) models are a flexible framework to control self-reported trait measurements for response styles. To this end, IRTree models decompose the responses to rating items into sub-decisions, which are assumed to be made on the basis of either the trait being measured or a response style, whereby the effects of such person parameters can be separated from each other. Here we investigate conditions under which the substantive meanings of estimated extreme response style parameters are potentially invalid and do not correspond to the meanings attributed to them, that is, content-unrelated category preferences. Rather, the response style factor may mimic the trait and capture part of the trait-induced variance in item responding, thus impairing the meaningful separation of the person parameters. Such a mimicry effect is manifested in a biased estimation of the covariance of response style and trait, as well as in an overestimation of the response style variance. Both can lead to severely misleading conclusions drawn from IRTree analyses. A series of simulation studies reveals that mimicry effects depend on the distribution of observed responses and that the estimation biases are stronger the more asymmetrically the responses are distributed across the rating scale. It is further demonstrated that extending the commonly used IRTree model with unidimensional sub-decisions by multidimensional parameterizations counteracts mimicry effects and facilitates the meaningful separation of parameters. An empirical example of the Program for International Student Assessment (PISA) background questionnaire illustrates the threat of mimicry effects in real data. The implications of applying IRTree models for empirical research questions are discussed.

Item response tree (IRTree) models are a popular class of multidimensional item response theory (IRT) approaches for analyzing self-reported Likert-type rating data (Böckenholt, 2012; De Boeck & Partchev, 2012). They rest on the assumption that item responding comprises several qualitatively distinct judgment steps, which are processed by respondents on the basis of different latent personal traits. A typical aim of using IRTree models is to separate the effects of the substantive trait to be measured from those of response styles, which are individual preferences for specific response categories of rating scales irrespective of item content (for an overview, see Van Vaerenbergh & Thomas, 2013). For instance, respondents may prefer extreme over non-extreme categories (extreme response style; ERS) or they tend to choose the middle categories of a scale (midscale response style; MRS). Since such different ways of using the rating scale can systematically bias trait estimates, there is great interest in both research and practice to apply methods that account for response styles and thereby provide valid trait measurements (Baumgartner & Steenkamp, 2001).

IRTree models provide an easy-to-implement framework for specifying various response styles in a theory-driven way. The ordinal responses to rating items are split into meaningful sub-decisions, which are modeled to be made on the basis of either the content-related trait or a response style. For example, respondents may first take a trait-based decision on whether they generally agree or disagree with the item, and subsequently select one of the available categories reflecting more or less intense agreement or disagreement driven by their response styles. Such sub-decisions are typically parameterized by unidimensional IRT models (e.g., the Rasch or 2PL model), so that the multidimensionality of IRTree models arises only between the sub-decisions, thus keeping the modeling complexity low and providing a straightforward interpretation of the parameters.

Several studies have demonstrated that IRTree models successfully capture multidimensional item responding, and such models were used for controlling trait measurements for response styles in various applications (e.g., Böckenholt & Meiser, 2017; Jeon & De Boeck, 2016; Khorramdel & von Davier, 2014; Kim & Bolt, 2021; Plieninger & Meiser, 2014; Tijmstra et al., 2018). However, the previous research solely focused on the assumption that response styles were actually involved in the item response process, so it is unclear how IRTree models perform in the absence of any response style effect. Even though the assignment of response styles to certain sub-decisions is theoretically founded, there may be circumstances in which respondents nevertheless make all their judgments solely on the basis of the trait; for example, if the respondents have a great interest in providing accurate information like in high-stakes assessments (e.g., personality assessments in job interviews). Since IRTree models are over-parameterized in such cases (i.e., they include several person parameters for modeling unidimensional data), they might be prone to overfitting and could carry the risk of estimating response style variance which is non-existent. Therefore, it remains to be investigated under which conditions the estimates obtained by IRTree modeling successfully reflect the substantive meaning assigned to the parameters and under which conditions they do not. Answering this question is of high relevance, as the potential lack of validity may compromise the key characteristic of IRTree models, which is their ability to disentangle the influences of multiple person parameters.

In addition, it is of particular concern that the estimated parameters labeled as response styles in misspecified IRTree models not only absorb random variance but may rather reflect trait-based responding and capture variance induced by the substantive trait. Since the response style factor then would mimic part of the trait, we call this methodological artifact a *mimicry effect*. The occurrence of such implies that the separability of traits and response styles is compromised, that is, the variance components in item responding are partially misattributed to a factor that is not the true source of the variance. Therefore, the mimicry effect is primarily manifested in a biased estimation of the relationship between trait and response style (i.e., their covariance and correlation). Furthermore, the variance of the response style factor is overestimated, as it captures additional trait-induced variance.

As a result, the pitfalls of mimicry effects in IRTree models for drawing conclusions from the data are twofold: First, the influences of content-unrelated category preferences are overestimated. Accordingly, even the dimensionality of the response process might be overestimated by an IRTree model if a response style was estimated to vary across respondents, despite not being part of the actual data-generating process. Second, the substantive meaning of the response style factor no longer corresponds to the meaning that was assigned to it, as the estimates at least partly reflect trait-based responding. Although the meanings of response styles and traits then overlap, they are considered distinct response processes given their associations with qualitatively different sub-decisions. Moreover, one might even find reasonable theoretical justifications for correlations between traits and given response styles post hoc (e.g., respondents with high levels of extraversion are likely to favor extreme response categories because they are generally self-confident), so that no further attention would be paid to an artificially induced covariance.

A likely scenario for an impaired separability of person parameters by IRTree models arises when the distribution of the respondents’ trait levels differs from that of the items of the questionnaire, such as when the trait follows a skewed or shifted distribution. For instance, skewed or shifted distributions are to be expected if the questionnaire was originally generated for a different sub-population of respondents with substantially higher or lower trait levels, or if a very rare or common trait is being assessed. An example would be questionnaires designed to measure the severity of mental disorders, for which the majority of the population scores low (e.g., the Beck Depression Inventory; Beck et al., 1996). Furthermore, many scales developed for the assessment of personality or attitudes of the general population have expected scores above the scale mean (e.g., five-factor models of personality like the International Personality Item Pool scales; Goldberg et al., 2006), while other scales have expected scores below the scale mean (e.g., the Dark Factor of Personality; Moshagen et al., 2018). All of these response patterns are likely the result of either skewed or shifted trait distributions in relation to the respective item distributions. Although it is thus inherently clear for a variety of empirical research questions that the distributions mismatch, there has been no systematic investigation of how this affects the parameter estimation and validity of IRTree models.

Therefore, the aim of this article is to evaluate the parameter estimation of IRTree models for various trait distributions with a focus on the separability of person parameters. Thereby, this article is intended to increase awareness for potentially biased estimates of response style parameters, in which case their assigned substantive meanings are invalid. Conversely, this does not mean that if person parameters are successfully separated by a model and statistically unbiased estimates are obtained, these estimates actually reflect the attributed substantive meaning in the sense of content validity. Yet, there is some evidence in the literature in favor of the validity of response style estimates: Investigations of the criterion validity of response styles showed that the IRTree estimates were linked to extraneous criteria as one would theoretically expect (Plieninger & Meiser, 2014; Zhang & Wang, 2020). In addition, individual response style estimates were found to be stable across different constructs (Wetzel et al., 2013) and over time (Weijters et al., 2010; Wetzel et al., 2016), which does not provide evidence for the validity per se, but still suggests that response styles are trait-like constructs and a characteristic of the persons rather than of the items or questionnaires. In a combined analysis of rating responses and response times, it was further revealed that responses that matched the person-specific response styles were faster, as one would expect given the conception of response styles as heuristic response processes (Henninger & Plieninger, 2020). Although these results support the use of IRT models accommodating response styles, such as IRTree models, the substantive validity of estimates can never be achieved without the accurate separation of traits and response styles. Therefore, with the analysis of the parameter separation in the present study, we are laying the groundwork for further investigations of the validity of IRTree models.

In the next section, IRTree models are formally introduced and the challenge of a meaningful separation of response style parameters from substantive traits is illustrated. Then, a series of three simulation studies is presented that examine the conditions under which IRTree models are at risk of compromised separability. Thereby, we quantify mimicry effects and explore how such a potential lack of validity can be detected. Since the main purpose of response style modeling in empirical research and practice is to obtain unbiased trait measurements, we additionally investigate the impact of mimicry effects on the recovery of the person-specific trait levels. In Simulation Study 1, the extent to which mimicry effects occur depending on the distribution of the substantive trait in relation to the items is assessed. In Simulation Study 2, potential remedies are evaluated with respect to their capability to counteract mimicry effects. As these two studies focus on the potential risk of applying IRTree models to data originating from a unidimensional response process (i.e., where the respondents’ decisions are purely trait-based), we investigate whether the findings are transferable to multidimensional data with the combined influences of trait and response styles in Simulation Study 3. Thereafter, an empirical application to the background questionnaire of the Program for International Student Assessment (PISA) 2018 study is presented, which demonstrates the threat of mimicry effects in real data. Finally, the results are discussed and implications for using IRTree models in empirical research are derived.

## Separation of Traits and Response Styles in IRTree Models

**Figure 1.**
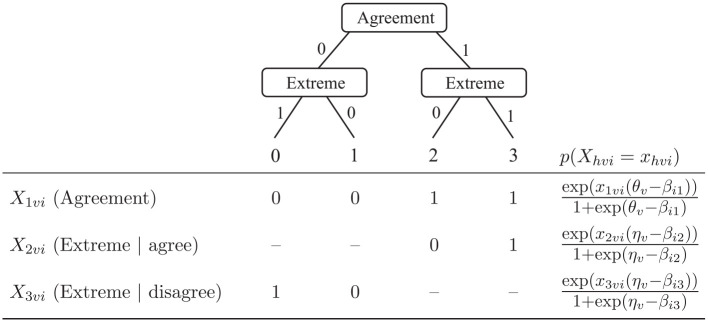
Tree Diagram and Definition of Pseudo-Items for Responses to 4-Point Rating Items. *Note*. Pseudo-items missing by design are marked with “–”.

IRTree models decompose the ordinal rating responses $Y_{\mathrm{vi}}\in \{0,...,K\}$ of person v=1,...,N to item i=1,...,I into a sequence of binary pseudo-items $X_{\mathrm{hvi}}$ , which represent the sub-decisions assumed to be taken by respondents during the response selection. The pseudo-items are usually parameterized by unidimensional IRT models of the trait or a response style, and the probability of an ordinal response is the product of the probabilities of responses to the respective pseudo-items. Figure 1 depicts a commonly used two-dimensional IRTree model for responding to items on a 4-point scale, with one sub-decision reflecting trait-based agreement, and a second one ERS-based extreme responding conditional on agreement. The pseudo-item responses are parameterized by Rasch models of either the substantive trait θ (h=1) or the ERS η (h=2andh=3) , and the probability of an ordinal response $Y_{\mathrm{vi}}\in \{0,...,3\}$ is obtained by

(1) $$p(Y_{\mathrm{vi}}=y_{\mathrm{vi}})=\left[\frac{\exp(x_{1\mathrm{vi}}(\theta_{v}-\beta_{i1}))}{1+\exp(\theta_{v}-\beta_{i1})}\right]\times {\left[\frac{\exp(x_{2\mathrm{vi}}(\eta_{v}-\beta_{i2}))}{1+\exp(\eta_{v}-\beta_{i2})}\right]}^{x_{1\mathrm{vi}}}\times {\left[\frac{\exp(x_{3\mathrm{vi}}(\eta_{v}-\beta_{i3}))}{1+\exp(\eta_{v}-\beta_{i3})}\right]}^{\left(1-x_{1\mathrm{vi}}\right)},$$

where $\beta_{\mathrm{ih}}$ denotes the difficulty of pseudo-item h of item i .

**Figure 2.**
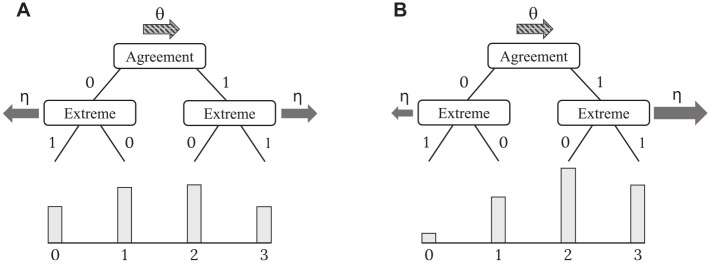
Illustration of the Meaningful Separation of Trait and ERS in an IRTree Model Depending on the Response Distribution. (A) Symmetrical Response Distribution. (B) Asymmetrical Response Distribution.

The separation of traits and response styles in IRTree models is achieved by defining model structures in which (a) the different personal characteristics are related to different pseudo-items (e.g., trait-based agreement and ERS-based extreme responding) and (b) they affect the selection of ordinal categories in unique ways that cannot be linearly transformed into each other (e.g., high trait levels favor high categories and high ERS levels favor extreme/outer categories). The first property leads to the identification of the model and enables the estimation of several parameters for each respondent. As these person parameters are assigned to different pseudo-items, a non-redundant part of the information from the ordinal responses is available for the estimation of each of them, and they can be statistically separated from each other. Nonetheless, only the second property ensures a meaningful distinction and substantive separation of different person parameters. Figure 2 illustrates the uniquely directed influences of trait and ERS for the IRTree model of 4-point rating items: Higher substantive trait levels are modeled to increase the probability of selecting agreement (i.e., higher) categories, whereas higher ERS levels favor the extreme categories, and thus either affect the response selection in the direction of the trait (i.e., higher categories conditional on agreement) or in the opposite direction (i.e., lower categories conditional on disagreement). The ERS factor is therefore assigned the substantive meaning of a preference for extreme categories based on its effects on the category selection across the two pseudo-items. It can only capture variance in the respondents’ behavior which equally affects the choice of extreme agreement and extreme disagreement categories.

Although such a definition of substantive traits and response styles as unique influences on different sub-decisions is theoretically reasonable, IRTree models may be misspecified such that the importance of a response style is being overstated or, correspondingly, the influence of the trait is being understated by the model. For instance, the true data-generating process could be a unidimensional one without any response style influence on the judgment process. Fitting an IRTree model assuming a response style influence (like the one depicted in Figure 1) to such unidimensional data entails the risk of a mimicry effect, as the response style parameters are not required to account for individual category preferences, and thus may be redeclared to capture variance which was actually introduced by the substantive trait. Such a redeclaration of the response style factor in terms of taking over part of the substantive trait would inevitably impede the meaningful separation of the two person parameters, though without affecting the statistical separation.

In the following, our hypotheses regarding the conditions of such an impairment of the meaningful separation of traits and response styles in IRTree models and the occurrence of mimicry effects are derived: Primarily, we expected that the statistical advantage of using response style parameters as a substitute for the substantive trait should depend on the distribution of the ordinal responses across the rating scale, which in turn is determined by the distribution of the substantive trait levels in relation to that of the items. If trait and item distributions match, the distribution of observed item responses is symmetrical (i.e., similar frequencies of agreement and disagreement categories; see Figure 2A), and a mimicry effect should not occur. In the exemplary 4-point IRTree model, the more the ERS factor would mimic the substantive trait, the better the variance among the agreement item responses should be accounted for, but the worse the variance among the disagreement categories. Thus, the congruent and opposing effects of trait and ERS should cancel out, they should have unique influences on the selection of ordinal categories, and their meaningful separation should remain intact.

In contrast, if the distribution of observed responses is asymmetrical (i.e., unequal distribution across agreement and disagreement categories; see Figure 2B), the congruent and opposing effects of trait and ERS can be assumed to not cancel out, and their influences on the selection of ordinal categories should partly overlap. A large proportion of the data should be better explained by ERS parameters mimicking the trait, and only a small proportion should be less well explained. Therefore, the model should benefit from a redeclaration of the ERS factor as a substitute for the trait, resulting in a substantial variance of the estimated ERS levels and covariance with the trait levels. Such a mimicry effect would be accompanied by a reduction or even complete loss of the meaningful separation of trait and response style, as the trait-induced variance would be jointly explained by both parameters. The severity of mimicry effects (i.e., the degree of reduction in separability) was thus assumed to be larger, the more asymmetrical the response distribution is, and accordingly, the more skewed or shifted the trait distribution in relation to the item distribution. Thereby, the reduced separation of trait and ERS was expected to occur for both an asymmetry of the response distribution toward high or low categories of the scale. However, an asymmetrical distribution toward higher categories should be associated with a positive covariance of ERS and trait, whereas a shift toward lower categories with a negative covariance. Since a negative covariance of two parameters implies the same degree of shared meaning as a corresponding positive one, the mimicry effect should be independent of the direction of the response distribution asymmetry and quantified by the absolute covariance.

In the next section, we illustrate our expectations regarding mimicry effects and their dependency on the distribution of rating responses in a first simulation study.

## Simulation Study 1—Mimicry Effects and Trait Distributions

The first simulation study addresses mimicry effects in IRTree models for various distributional conditions of the trait in the absence of response style influences. Therefore, unidimensional item response data were generated under the partial credit model (PCM; Masters, 1982), which was chosen as the data-generating model as it is a commonly used IRT model for ordinal rating data. The two-dimensional IRTree model with trait and ERS influences illustrated in Figure 1 was used as the analysis model. The underlying trait distributions were set to be either skewed or shifted in relation to the items, which both result in asymmetrical response distributions. Besides the investigation of mimicry effects, we evaluated the recovery of the substantive trait levels, which provides an indication of whether IRTree models produce reasonable estimates for individual parameters despite the risk of a biased estimation of the covariance structure.^1^

### Simulation Design

Item response data were generated under the PCM, a unidimensional IRT model in which the selection of all ordinal response categories is assumed to depend solely on the substantive trait. The category probabilities of the ordinal responses $Y_{\mathrm{vi}}\in \{0,...,K\}$ under the PCM are given by

(2) $$p(Y_{\mathrm{vi}}=y_{\mathrm{vi}})=\frac{\exp\left(y\theta_{v}-\sum_{k=0}^{y}\beta_{\mathrm{ik}}\right)}{\sum_{j=0}^{K}\exp\left(j\theta_{v}-\sum_{k=0}^{j}\beta_{\mathrm{ik}}\right)},$$

with $\beta_{i0}:=0$ . $\theta_{v}$ denotes the person-specific trait level and $\beta_{\mathrm{ik}}$ denotes the item- and category-specific difficulty. The difficulty parameters can be decomposed as $\beta_{\mathrm{ik}}=\beta_{i}+\tau_{\mathrm{ik}}$ , where $\beta_{i}$ denotes the item location and is defined as $\sum_{k=1}^{K}\beta_{\mathrm{ik}}/K$ and $\tau_{\mathrm{ik}}$ denotes the category-specific deviations with $\sum_{k=1}^{K}\tau_{\mathrm{ik}}=0$ .

Responses to 4-point rating items were generated, with the category-specific difficulty parameters $\beta_{\mathrm{ik}}$ for each item drawn from a uniform distribution U(−3,3) and assigned to the ordinal categories k={1,2,3} in ascending order ( $\beta_{i0}$ is defined to be 0 in the PCM). This sampling procedure results in the item locations $\beta_{i}$ being approximately normally distributed with mean 0 and variance 1.

For conditions of shifted trait distributions, person-specific trait levels $\theta_{v}$ were sampled from normal distributions N(μ,1.0) with mean μ set to 0.0, 0.2, 0.5, or 1.0. Therefore, the distributions of the traits and item locations either matched ( μ=0.0 ; baseline condition) or were shifted by 0.2, 0.5, or 1.0 units of the SD . The stronger the shifts of the trait distributions, the more the distributions of the ordinal item responses were asymmetrical toward the higher categories of the scale.

For the skewed conditions, the substantive trait was assumed to stem from a skew-normal distribution with the probability density function:

(3) $$\mathrm{SkewN}(x\;|\;\xi ,\omega ,\alpha )=\frac{2}{\omega }\phi \left(\frac{x-\xi }{\omega }\right)\Phi \left(\alpha \left(\frac{x-\xi }{\omega }\right)\right),$$

where ϕ denotes the standard normal probability density function and Φ denotes the cumulative distribution function (for further details on the skew-normal distribution, see Azzalini & Capitanio, 2014). The parameter ξ is the location, ω is the scale, and α is the skewness of the distribution. Positive α values result in right-skewed distributions and negative values in left-skewed ones. The skew-normal distribution reduces to the standard normal one for ξ=0 , ω=1 , and α=0 . The mean and variance of a skew-normally distributed variable X~SkewN(ξ,ω,α) are defined as

(4) $$M=\xi +\frac{\omega \alpha \sqrt{2}}{\sqrt{(1+a^{2})\pi })},$$

(5) $$\mathrm{Var}=\omega^{2}\left(1-\frac{2\alpha^{2}}{\pi (1+\alpha^{2})}\right).$$

The trait levels were sampled from SkewN(ξ=0,ω,α) , with skewness parameter α set to 0.0, 0.5, 1.0, and 2.0. The corresponding scale parameters ω were set to 1.00, 1.07, 1.21, and 1.43, resulting in the trait distributions of all conditions having a variance of 1, which provided a high degree of comparability between all shifted and skewed conditions. According to Equation 4, the means of the four conditions were 0.00, 0.38, 0.68, and 1.02. The baseline condition with skewness parameter α=0.0 was equivalent to sampling from a standard normal distribution and thus equivalent to the baseline condition of the shifted data generation. The higher the skewness parameters were, the stronger the asymmetry of ordinal item responses toward the higher categories of the scale.

For each of the four shifted and four skewed conditions, random data sets were generated with varying sample sizes N , set to 100, 500, and 2,000, and questionnaire lengths I , set to 5, 10, 20, and 40. The simulation factors were fully crossed, resulting in 96 (8 × 3 × 4) simulation conditions, for which 100 replications were conducted each. For each data set, item responses were generated as follows:^2^ N trait levels and 3×I item difficulties were randomly drawn according to the sampling procedure of the respective simulation condition described above. Then, person and item parameters were inserted into the PCM given by Equation 2, yielding category-specific probabilities for responses of each person to each item. Finally, ordinal item responses were sampled according to the model-implied probabilities, and pseudo-item responses were derived from such ordinal responses according to the definition given in Figure 1.

The generated data sets were analyzed within the IRTree framework, and the model described in Figure 1 and Equation 1 was applied. We additionally used the PCM as an analysis model to obtain benchmarks for the trait recovery. The models were estimated using the R package *mirt*^3^ (Chalmers, 2012) and all models converged.

The recovery of substantive traits was assessed by the correlation of generated and estimated (expected a posteriori) parameters. This measure of recovery was chosen as it indicates whether the ranking of the persons was correctly reflected by the model estimates. Other commonly used measures of recovery, such as the mean absolute bias, were not suitable here as the conditions with shifted or skewed trait distributions necessarily result in larger absolute deviations of estimated parameters. In addition, the rank order is a crucial measure when assessments are used as the basis for decisions in a practical context, such as the selection of the best applicants in a job interview.

Further note that for the data generation under both shifted and skewed trait distributions, only conditions resulting in an asymmetrical response distribution toward the higher categories were defined. We chose such conditions since asymmetrical distributions in the opposite direction toward low categories can be expected to not affect the size of the mimicry effects in IRTree models which have a symmetrical tree structure. The IRTree model used here has such a symmetrical structure, as the agreement sub-decision splits the rating scale into two categories each, which are then again split by the extreme sub-decisions. Thus, we assumed that the only difference in the mimicry effects for asymmetrical response distributions toward high and low categories should be that the deviation of the estimated covariance from the true covariance reverses in sign. We nevertheless run the same simulation study as described here just with reversed trait shifts (μ=−0.2,−0.5,or−1.0) and reversed skewness parameters ( α = −0.5, −1.0, and −2.0). The results can be found in the Online Supplementary Materials and confirm our assumption that the corresponding positive and negative parameters resulted in nearly equivalent sizes of the mimicry effects. Therefore, only conditions associated with an asymmetry to the high categories are presented in the following.

### Results

The mimicry effects were evaluated by the estimated covariances between ERS and substantive trait levels. As the data-generating process was unidimensional and did not incorporate response style influences, the accurate estimate for the covariance would be zero. Thus, the more an estimated covariance deviated from zero, the stronger the mimicry effect was. In line with our expectations, the mimicry effects were more pronounced the more skewed or shifted the underlying trait distributions were in relation to the distribution of item locations (i.e., the more asymmetrical the item response distributions), as can be seen in Table 1. Likewise, the estimated correlations between ERS and trait strongly increased with increasingly asymmetric response distributions. For the baseline conditions with non-shifted or non-skewed distributions, the covariances and correlations were correctly estimated to be close to zero.

**Table 1.** Estimated Covariances, Correlations, and Variances of ERS η and Trait θ by the IRTree Model for Unidimensional Data (Simulation 1).

Trait distribution		*M* (*SD*) across replications			
Distr. family	Condition	$\hat{\mathrm{Cov}}(\eta ,\theta )$	$\hat{\mathrm{Cor}}(\eta ,\theta )$	$\hat{\mathrm{Var}}(\eta )$	$\hat{\mathrm{Var}}(\theta )$
Shifted	μ=0.0	0.00 (0.26)	0.00 (0.34)	0.26 (0.13)	2.11 (0.37)
	μ=0.2	0.15 (0.26)	0.20 (0.34)	0.26 (0.12)	2.11 (0.37)
	μ=0.5	0.35 (0.27)	0.44 (0.29)	0.31 (0.15)	2.14 (0.36)
	μ=1.0	0.70 (0.26)	0.74 (0.18)	0.40 (0.16)	2.29 (0.43)
Skewed	α=0.0	0.01 (0.26)	0.01 (0.34)	0.26 (0.14)	2.12 (0.39)
	α=0.5	0.28 (0.26)	0.36 (0.30)	0.28 (0.13)	2.15 (0.38)
	α=1.0	0.54 (0.27)	0.62 (0.25)	0.35 (0.15)	2.18 (0.39)
	α=2.0	0.84 (0.23)	0.83 (0.13)	0.49 (0.18)	2.14 (0.41)

*Note.* Aggregated across sample sizes (N=100,500,2,000) and questionnaire lengths (I=5,10,20,40) . ERS = extreme response style.

The conditions with shifted and skewed trait distributions hardly differed with respect to revealing increasing mimicry effects for increasing deviations from the standard normal baseline condition. However, the skewed conditions generally yielded slightly stronger effects, suggesting that the asymmetry of item responses was higher for the specific skewness parameters α , compared with the specific mean shifts μ we defined. Sample size and questionnaire length did not influence the mimicry effect, as there were only small differences in the estimated covariances across these simulation factors (see Table A1 in the Online Supplementary Materials).

Furthermore, the estimated variances of the ERS factor increased with higher shifts or skewness parameters of the trait distributions, which is in line with the assumption that the ERS parameters increasingly take over trait-induced variance for a stronger asymmetry of observed item responses. Also for the baseline conditions without mimicry effects, the ERS variances were greater than zero, indicating that the parameters still captured some variation in the selection of extreme categories across respondents. Nevertheless, even in conditions with strong mimicry effects, the average variance estimates of the ERS factor were rather small in comparison to those of the trait, which is due to the fact that the data-generating process was purely trait-based and did not include ERS-based responding. Furthermore, the estimated trait variances were much higher than the generated variances of 1, which is due to differences in the model specifications between the data-generating PCM and the analysis IRTree model.

Overall, the analysis of the variance and covariance estimation clearly demonstrated that IRTree models pose the risk of mimicry effects, and thus potentially lead to inaccurate conclusions about the involved response processes and the relationship of the person parameters. Even for trait distributions with slight shifts or small degrees of skewness, the estimated covariances of trait and ERS were of substantial size. Such estimates would likely lead researchers to the erroneous interpretation that respondents with high levels of the substantive trait had strong preferences for extreme categories and those with low trait levels rather preferred the non-extreme ones, when in fact, the respondents did not at all have category preferences.

However, since a main use case of IRTree modeling is to obtain accurate trait measurements that are controlled for response style influences, the response style estimates themselves or covariances with other parameters are often not of interest. Therefore, we additionally examined the recovery of the substantive trait levels. Notably, the presence of a mimicry effect did not impair the trait recovery by the IRTree model. Irrespective of the distributional condition, the correlations of generated and estimated trait levels were consistently high, as is evident from Table 2 (also see Table A2 in the Online Supplementary Materials for the trait recovery split by N and I ). The PCM yielded a slightly higher trait recovery in all conditions, which can be considered the benchmark or maximal achievable values of recovery, as the PCM was the true data-generating model. However, the PCM uses the information of all four ordinal response categories for the estimation of the trait levels, while the IRTree model only uses the information provided by the binary agreement sub-decision, so this small advantage of the PCM is not surprising.

**Table 2.** Trait Recovery Cor(θ , $\hat{\theta })$ by the IRTree Model and Data-Generating PCM for Unidimensional Data (Simulation 1).

Trait distribution		*M* (*SD*) across replications	
Distr. family	Condition	IRTree	PCM
Shifted	μ=0.0	0.87 (0.08)	.91 (.06)
	μ=0.2	0.87 (0.08)	.91 (.06)
	μ=0.5	0.88 (0.08)	.91 (.06)
	μ=1.0	0.88 (0.07)	.91 (.06)
Skewed	α=0.0	0.88 (0.07)	.91 (.06)
	α=0.5	0.88 (0.07)	.91 (.06)
	α=1.0	0.88 (0.07)	.91 (.06)
	α=2.0	0.88 (0.08)	.90 (.06)

*Note.* Aggregated across sample sizes (N=100,500,2,000) and questionnaire lengths (I=5,10,20,40) . *SD* = standard deviation; PCM = partial credit model.

Thus, the potential occurrence of a mimicry effect in IRTree modeling is primarily a concern for estimating response styles, but less so for recovering person-specific trait levels. If the focus of the analysis is exclusively on the measurement of substantive traits, our results suggest that skewed or shifted trait distributions do not have relevant effects. Nonetheless, a bias in the latent covariance matrix may lead to misinterpretations regarding the item response process and involved person parameters. Therefore, we explore possible modifications of the previously used IRTree model in the next section, which potentially could counteract mimicry effects and provide unbiased estimates of all model parameters.

## Simulation Study 2—Modified IRTree Models Counteracting Mimicry Effects

In the second simulation study, two modified IRTree models were examined with regard to their ability to counteract mimicry effects. The first modified IRTree model differed from the standard IRTree model described before (see Figure 1 and Equation 1) in that the covariance of trait and ERS was fixed to zero. This model constraint prevents the estimation of artificial covariances, as evoked by the ERS parameters mimicking the substantive traits. Consequently, erroneous conclusions about the relationship between the trait and response styles cannot arise even in the presence of asymmetrical response distributions. However, this comes with the disadvantage that such a model cannot capture a true covariance if it would actually be present in the data, and that a zero covariance of personal characteristics is often not reasonable from a theoretical point of view.

Therefore, another modified IRTree model with freely estimated covariance was evaluated, in which the extreme pseudo-items were parameterized by multidimensional IRT models (see Böckenholt, 2019; Jeon & De Boeck, 2016; Meiser et al., 2019). Such a multidimensionality within pseudo-items (additionally to the usual multidimensionality between pseudo-items) reflects the assumption that not only one, but several person parameters are involved in the respective sub-decisions. For instance, in the IRTree model for 4-point rating items, respondents may use both the ERS and the trait for the sub-decisions of extreme versus non-extreme responding. Previous studies showed that, indeed, response styles and traits are often simultaneously involved in certain sub-decisions in empirical data (Meiser et al., 2019; Merhof & Meiser, 2023; von Davier & Khorramdel, 2013). Moreover, multidimensional pseudo-items have the advantage that even if the sub-decisions originate from a unidimensional response process, it is not required to specify in advance which person parameter is driving this decision. Rather, both the dimensionality of sub-decisions and the involved parameters can be explored in the given data.

The IRTree model with multidimensional pseudo-items used in the following is given by

(6) $$\begin{array}{c} p(Y_{\mathrm{vi}}=y_{\mathrm{vi}})=\left[\frac{\exp(x_{1\mathrm{vi}}(\theta_{v}-\beta_{i1}))}{1+\exp(\theta_{v}-\beta_{i1})}\right]\times {\left[\frac{\exp(x_{2\mathrm{vi}}(\eta_{v}+\lambda \theta_{v}-\beta_{i2}))}{1+\exp(\eta_{v}+\lambda \theta_{v}-\beta_{i2})}\right]}^{x_{1\mathrm{vi}}}\\ \;\;\;\;\;\;\;\;\;\;\;\;\;\;\;\times {\left[\frac{\exp(x_{3\mathrm{vi}}(\eta_{v}-\lambda \theta_{v}-\beta_{i3}))}{1+\exp(\eta_{v}-\lambda \theta_{v}-\beta_{i3})}\right]}^{\left(1-x_{1\mathrm{vi}}\right)}. \end{array}$$

with λ≥0 .

The model differs from the standard IRTree model with unidimensional pseudo-items only in the parameterization of extreme responding, for which in addition to the ERS η , also the trait θ is assumed to influence the respondents’ decisions. The weight parameter λ indicates the relative importance of the trait for extreme responding in relation to its importance for the agreement decisions, in which it is weighted by one. The trait is given opposite signs for extreme responding conditional on the agreement judgment to account for the fact that extreme agreement is more likely under both high ERS and high trait levels of respondents, whereas the probability of selecting extreme instead of non-extreme disagreement still increases with higher ERS but decreases with higher trait levels. These differently directed influences of trait and ERS across the extreme pseudo-items facilitate the statistical and meaningful separation of the two person parameters, despite the fact that they do not relate to distinct sub-decisions.

IRTree models with multidimensional pseudo-items can be expected to counteract mimicry effects since the response style parameters are statistically ineffective substitutes for the substantive trait if the trait itself is also included in the respective sub-decisions. As illustrated previously for the IRTree model with unidimensional parameterization (see Figure 2), ERS parameters mimicking the trait are advantageous for explaining the trait-induced variance of extreme responding for one side of the rating scale (e.g., variance among the agreement categories) and disadvantageous for the other side (e.g., variance among the disagreement categories). Only if the responses are asymmetrically distributed over both sides of the rating scale, as is the case for shifted or skewed trait distributions, the model benefits from the redeclaration of the ERS factor. In contrast, since the IRTree model with multidimensional pseudo-items incorporates trait influences for all sub-decisions, the trait parameters can account for the trait-induced variance in extreme responding independently of the response distribution. Multidimensional pseudo-items can thus be assumed to not only maintain the statistical separation of traits and response styles but also enhance the meaningful separation of such parameters in comparison to the unidimensional parameterization.

In the second simulation study, both the IRTree model with multidimensional pseudo-items and the model with fixed covariance were evaluated with regard to mimicry effects and trait recovery.^3^ They were compared against the standard IRTree model used in the first simulation study, in which the covariance of trait and ERS was estimated and all pseudo-items were parameterized by unidimensional IRT models. The same unidimensional data-generating procedure by the PCM as in the first simulation study was applied. As mimicry effects were found to likewise occur for data with shifted and skewed trait distributions (see Table 1), only the shifted conditions were considered here. Furthermore, sample size and questionnaire length were not varied ( N was set to 500, and I was set to 20), as no relevant differences were observed (see Tables A1 and A2 in the Online Supplementary Materials). 100 replications were conducted for each shifted condition with μ set to 0.0, 0.2, 0.5, and 1.0.

### Results

The analysis of the estimated variances and covariances (see Table 3) revealed that the IRTree model with fixed covariance was only suitable to a limited extent in terms of counteracting mimicry effects and the misattribution of trait-induced variance. Although fixing the covariances of ERS and trait naturally prevents mimicry effects in the strict sense, the estimated variances of ERS parameters increased with increasing trait shifts. This overestimation of the ERS variance suggests that the ERS parameters still captured part of the trait-induced variance in extreme responding. We therefore investigated whether, despite the zero-constrained population covariance $\left(\hat{\mathrm{Cov}}(\eta ,\theta )=0\right)$ , the covariance of the estimated trait and ERS levels $\left(\mathrm{Cov}(\hat{\eta },\hat{\theta })\right)$ nevertheless differed from zero. The covariance of estimated parameters indeed increased with increasing trait shifts, demonstrating that a kind of hidden mimicry effect occurred. As a result, the ERS parameters mimicked the trait, causing them to covary with each other, although the constrained population covariance supposedly specified that there was no relationship between the parameters. As this hidden mimicry effect was smaller compared with the actual mimicry effect that occurred in the standard IRTree model, forcing the population covariance to zero seems to have suppressed at least part of the redeclaration of the ERS parameters (for μ = 1.0, the hidden effect was 0.26 and the mimicry effect of the standard IRTree model was 0.69, see Table A1 in the Online Supplementary Materials, condition with N = 500, I = 20). Nevertheless, the model with fixed covariance did not prevent biases in the parameter estimation to a satisfactory degree, as it still indicated that an ERS influence was present, even though it was not part of the data-generating process.

**Table 3.** Estimated Covariances, Correlations, and Variances of ERS η and Trait θ by the Modified IRTree Models for Unidimensional Data (Simulation 2).

Analysis	Trait shift	*M* (*SD*) across replications				
		$\hat{\mathrm{Cov}}(\eta ,\theta )$	$\mathrm{Cov}(\hat{\eta },\hat{\theta })$	$\hat{\mathrm{Cor}}(\eta ,\theta )$	$\hat{\mathrm{Var}}(\eta )$	$\hat{\mathrm{Var}}(\theta )$
IRTree fixed covariance	μ=0.0	0.00 (0.00)*	0.00 (0.07)	0.00 (0.00)*	0.24 (0.06)	2.09 (0.20)
	μ=0.2	0.00 (0.00)*	0.05 (0.07)	0.00 (0.00)*	0.24 (0.06)	2.10 (0.23)
	μ=0.5	0.00 (0.00)*	0.12 (0.07)	0.00 (0.00)*	0.26 (0.07)	2.10 (0.22)
	μ=1.0	0.00 (0.00)*	0.26 (0.09)	0.00 (0.00)*	0.34 (0.08)	2.13 (0.22)
IRTree multidimensional	μ=0.0	−0.01 (0.06)	−0.01 (0.05)	−0.05 (0.18)	0.04 (0.01)	2.10 (0.20)
	μ=0.2	−0.02 (0.06)	−0.02 (0.05)	−0.08 (0.19)	0.04 (0.01)	2.10 (0.23)
	μ=0.5	−0.02 (0.06)	−0.01 (0.05)	−0.06 (0.20)	0.04 (0.01)	2.10 (0.24)
	μ=1.0	−0.05 (0.07)	−0.03 (0.06)	−0.14 (0.20)	0.05 (0.01)	2.13 (0.23)

*Note.* N=500 , I=20 . ERS = extreme response style; *SD* = standard deviation.

* Covariance and correlation of ERS and trait are not estimated but fixed to zero.

In contrast, the IRTree model with multidimensional pseudo-items provided estimates of the ERS variance which were very close to zero regardless of the trait distribution. Thus, it successfully detected the unidimensional data-generating process and accurately reflected the absence of response style influences. The covariances of ERS and trait were likewise correctly estimated to be close to zero so that mimicry effects did not occur. The IRTree model with multidimensional pseudo-items therefore consistently prevented a misattribution of the trait-induced variance even for strongly asymmetrical response distributions.

Somewhat unexpectedly, the correlations of the ERS with the trait estimated by the model with multidimensional pseudo-items were on average slightly negative. As these estimates largely varied across simulation replications, and given that the individual ERS factors had a very low variance, this correlation is likely an artifact of the estimation and suggests that the parameters adapted to small random variations of the respondents’ selection of extreme categories. Furthermore, the fact that the variance of the ERS and its covariance with the trait were consistently estimated to be close to zero for all data sets, the model would hardly mislead to the false interpretation that the small correlations between trait and ERS were substantially meaningful.

Also, the recovery of trait levels (see Table 4) was comparatively better in the IRTree model with multidimensional pseudo-items than in the models with unidimensional ones (with fixed or estimated covariance) and even reached the benchmark recovery by the true data-generating PCM. However, the models with unidimensional pseudo-items performed only slightly worse and still yielded satisfactory recovery. As was shown in the first simulation study, mimicry effects and misspecifications of IRTree models were only of limited relevance for the trait recovery and were more severe in terms of possible incorrect conclusions about the presence and importance of response styles.

**Table 4.** Trait Recovery Cor(θ , $\hat{\theta })$ for Unidimensional Data (Simulation 2).

Trait shift	*M* (*SD*) across replications			
	IRTree	IRTree fixed coverage	IRTree multidimensional	PCM
μ=0.0	0.92 (0.01)	0.92 (0.01)	0.95 (0.01)	0.95 (0.01)
μ=0.2	0.92 (0.01)	0.92 (0.01)	0.95 (0.01)	0.95 (0.01)
μ=0.5	0.92 (0.01)	0.92 (0.01)	0.95 (0.00)	0.95 (0.00)
μ=1.0	0.92 (0.01)	0.91 (0.01)	0.94 (0.01)	0.94 (0.01)

*Note.* N=500 , I=20 . *SD* = standard deviation; PCM = partial credit model.

Altogether, the second simulation study demonstrated that the model with multidimensional pseudo-items successfully counteracted mimicry effects and further recovered the substantive trait levels very well. These results suggest that a multidimensional parameterization of pseudo-items should be preferred to a unidimensional one if it seems plausible from a theoretical perspective, for instance, if sub-decisions that are assumed to be based on response styles may be additionally influenced by the trait. So far, though, we have provided evidence for the benefits of the multidimensional parameterization only for unidimensional data. However, item responding without any response style influence is (a) hardly found in empirical data and (b) contrary to the primary purpose of using IRTree models, namely to control trait measurements for response style effects. Therefore, a third simulation study was conducted, in which the previously analyzed IRTree models with unidimensional or multidimensional pseudo-items were fitted to data originating from a multidimensional response process with ERS influence.

## Simulation Study 3—Multidimensional Data With Response Style Influence

The third simulation study concerned mimicry effects in multidimensional item response data, for which, in addition to the trait, also a response style was assumed to affect the selection of rating categories. For such data, the mimicry effect is the difference between the true and estimated covariances, which is in contrast to the first two studies where the estimated covariance directly quantified the mimicry effect. Nevertheless, just as for unidimensional data, we assumed that the response style parameters should mimic the trait and capture part of its variance if a misspecified IRTree model overstated the influence of a response style and understated the influence of the trait. For example, an IRTree model could suggest that the extreme sub-decisions were purely ERS-based, although, in fact, the trait additionally affected the response selection. Since a skewed or shifted trait distribution results in an asymmetrical response distribution also for multidimensional data, we expected a statistical advantage of adjusting the meaning of the ERS toward that of the substantive trait. However, unlike in unidimensional data, only part of the estimated response style variance should then reflect trait-based responding, and the other part should reflect actual differences in individual category preferences. In the data example illustrated in Figure 2B, the person-specific ERS estimates should thus represent a compromise between the true preferences for extreme categories and trait-based responding. The balance of this compromise should depend on the response distribution so that a higher asymmetry should cause the estimated ERS levels to more closely reflect the substantive meaning of the trait. Likewise, the estimated covariance of response style and trait should comprise the sum of both the true relationship of the latent personal characteristics and the artificially evoked covariance. Thereby, the direction of the asymmetry can be assumed to determine whether the covariance is overestimated or underestimated, so a mimicry effect may even change the sign of the estimated relationship.

Our hypotheses on mimicry effects in multidimensional data were tested in the simulation study by generating item response data under the IRTree model with multidimensional pseudo-items according to Equation 6, in which the agreement sub-decision is modeled to be solely dependent on the trait, and the extreme sub-decisions are parameterized by both the trait and the ERS.^2^ The parameter λ , which indicates the importance of the trait for extreme responding, was set to 0.5 across all generated data sets, as previous studies showed that such is a realistic value for empirical data (Meiser et al., 2019; Merhof & Meiser, 2023). Since the mimicry effect is the covariance of a response style and the trait which deviates from the true relationship of these two parameters, we varied the covariance of ERS and trait as an additional simulation factor and set it to 0.0, 0.2, 0.4, and 0.6. The variances of both ERS and trait were set to 1, so the generated covariances corresponded to the correlations. The trait shift μ was varied and set to 0.0 and 1.0. The sample size N was set to 100, 500, and 2,000; the questionnaire length I was set to 5, 10, 20, and 40. 100 replications were conducted for each condition of the fully crossed simulation factors. The analysis models were the standard IRTree model with unidimensional pseudo-items, the IRTree model with multidimensional pseudo-items, and the PCM.

### Results

The analyses clearly demonstrated that mimicry effects also occur if a response style was involved in the data-generating process in addition to the trait. As shown in Table 5, the standard IRTree model with unidimensional pseudo-items yielded inflated estimates of the covariance in case of a trait shift. The true covariance of ERS and trait hardly influenced the mimicry effect, as the overestimation of covariance was consistent across the conditions of generated covariances. However, with an average overestimation of 0.14, the mimicry effect was considerably smaller than the corresponding effect for unidimensional data (0.70, see Table 1). This difference is due to the fact that the ERS parameters only capture the trait-induced variance of extreme responding for unidimensional data but are a compromise of the trait and actual ERS levels of respondents in multidimensional data. As was shown for unidimensional data, sample size and questionnaire length did not influence the mimicry effect (see Table A3 in the Online Supplementary Materials).

**Table 5.** Estimated Covariances and Correlations of ERS η and Trait θ for Multidimensional Data With ERS Influence (Simulation 3).

Analysis	Cov( η , θ )	*M* (*SD*) across replications			
		$\hat{\mathrm{Cov}}(\eta ,\theta )$		$\hat{\mathrm{Cor}}(\eta ,\theta )$	
		μ=0.0	μ=1.0	μ=0.0	μ=1.0
IRTree	0.0	0.00 (0.16)	0.14 (0.16)	0.00 (0.15)	0.14 (0.16)
	0.2	0.19 (0.16)	0.33 (0.16)	0.19 (0.16)	0.32 (0.14)
	0.4	0.38 (0.16)	0.54 (0.17)	0.38 (0.14)	0.51 (0.13)
	0.6	0.57 (0.17)	0.73 (0.17)	0.58 (0.13)	0.67 (0.10)
IRTree multidimensional	0.0	−0.01 (0.14)	−0.01 (0.14)	−0.01 (0.20)	0.00 (0.19)
	0.2	0.19 (0.14)	0.19 (0.14)	0.20 (0.20)	0.21 (0.18)
	0.4	0.40 (0.14)	0.40 (0.14)	0.42 (0.17)	0.43 (0.18)
	0.6	0.59 (0.15)	0.59 (0.15)	0.62 (0.13)	0.63 (0.13)

*Note.* Aggregated across sample sizes (N=100,500,2,000) and questionnaire lengths (I=5,10,20,40) . ERS = extreme response style.

The IRTree model with multidimensional pseudo-items again proved to be resistant to the mimicry effect, as it provided unbiased estimates of the covariance across all conditions also for multidimensional data. This finding corroborates our previous suggestion that a multidimensional parameterization of pseudo-items should be preferred to a unidimensional one if unidimensionality is not required from a theoretical point of view. Also in terms of person parameter recovery, a similar pattern to that observed for unidimensional data was found: The differences in recovery of trait and ERS levels between the models were small, with a slight advantage of the true data-generating IRTree model with multidimensional pseudo-items (see Table 6). Only in conditions with few data points ( N=100 and I=5 ), the recovery by the IRTree model with multidimensional pseudo-items was slightly worse compared with the other models, which is probably due to the greater complexity of this model (see Tables A4 and A5 in the Online Supplementary Materials for the recovery of person parameters split by N and I ).

**Table 6.** Parameter Recovery for Multidimensional Data With ERS Influence (Simulation 3).

Analysis	Cov( η , θ )	*M* (*SD*) across replications			
		Cor( θ , $\hat{\theta }$ )		Cor( η , $\hat{\eta }$ )	
		μ=0.0	μ=1.0	μ=0.0	μ=1.0
IRTree	0.0	0.80 (0.11)	0.79 (0.12)	.78 (.11)	.77 (.11)
	0.2	0.80 (0.11)	0.79 (0.11)	.78 (.11)	.78 (.11)
	0.4	0.80 (0.11)	0.80 (0.10)	.79 (.11)	.79 (.10)
	0.6	0.81 (0.10)	0.82 (0.09)	.80 (.10)	.81 (.09)
IRTree multidimensional	0.0	0.82 (0.11)	0.81 (0.11)	.78 (.13)	.78 (.14)
	0.2	0.82 (0.12)	0.81 (0.11)	.78 (.13)	.79 (.12)
	0.4	0.82 (0.11)	0.82 (0.11)	.79 (.12)	.79 (.12)
	0.6	0.83 (0.10)	0.84 (0.09)	.81 (.11)	.81 (.11)
PCM	0.0	0.81 (0.10)	0.80 (0.10)	—	—
	0.2	0.81 (0.10)	0.81 (0.10)	—	—
	0.4	0.81 (0.10)	0.82 (0.10)	—	—
	0.6	0.81 (0.10)	0.82 (0.09)	—	—

*Note.* Aggregated across sample sizes (N=100,500,2,000) and questionnaire lengths (I=5,10,20,40) . ERS = extreme response style; *SD* = standard deviation; PCM = partial credit model.

## Application

To demonstrate the impact of mimicry effects on the validity of conclusions drawn from empirical data, two scales of the background questionnaire of the PISA 2018 study were analyzed by IRTree modeling. We used the item responses of N=4,411 participants to the 2 scales “reading self-evaluation” comprising 6 items and “reading enjoyment” comprising five items on a 4-point rating scale.^4^ The subset of the data considered here is described in more detail by Henninger and Meiser (2023). The standard IRTree model with unidimensional pseudo-items as well as the IRTree model with multidimensional pseudo-items were fitted to the data. As the multidimensional model was shown to produce unbiased estimates in the simulation studies, it was considered the benchmark model with which the standard IRTree model was compared in order to quantify mimicry effects.

First, both scales were analyzed separately for illustration purposes. The results are summarized in Table 7 and suggest mimicry effects in the standard IRTree model for both scales: The estimated covariances, correlations, and ERS variances largely differed between the two models, indicating a biased estimation by the IRTree model with unidimensional pseudo-items. For “reading self-evaluation,” the correlation of ERS and trait under the standard IRTree model was of substantial size, which was strongly reduced when the model with multidimensional pseudo-items was applied. For “reading enjoyment,” a mimicry effect was likewise apparent, though in the opposite direction: The model with unidimensional pseudo-items indicated that trait and ERS levels were unrelated, when in fact the model with multidimensional pseudo-items showed that they were positively correlated. In both cases, the larger ERS variances in the standard IRTree model supported the presence of mimicry effects.

**Table 7.** Estimated Covariances, Correlations, and Variances of ERS η and Trait θ for the Empirical PISA Data.

PISA scale	Analysis	$\hat{\mathrm{Cov}}(\eta ,\theta )$	$\hat{\mathrm{Cor}}(\eta ,\theta )$	$\hat{\mathrm{Var}}(\eta )$	$\hat{\mathrm{Var}}(\theta )$
Reading self-evaluation	IRTree	1.92	0.41	5.74	3.87
	IRTree multidimensional	0.72	0.19	4.02	3.63
Reading enjoyment	IRTree	0.12	0.02	3.38	7.10
	IRTree multidimensional	0.49	0.13	2.16	7.05

*Note.* ERS = extreme response style; PISA = Program for International Student Assessment.

**Figure 3.**
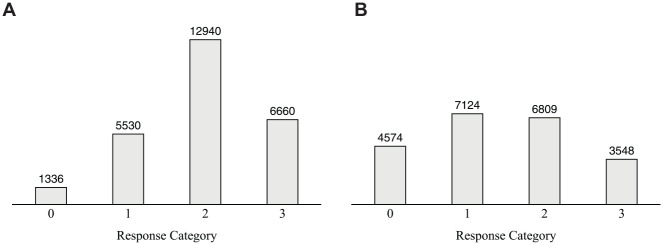
*Response Distributions and Absolute Category Frequencies Across the Items of the Two Scales in the Empirical PISA Data. (A) Reading Self-Evaluation. (B) Reading Enjoyment*. *Note*. PISA = Program for International Student Assessment.

Thereby, the distributions of the observed item responses (see Figure 3) further demonstrated that even a slight asymmetry toward one side of the rating scale can distort the interpretation of the results derived from the IRTree model with unidimensional pseudo-items: Whereas the item responses of the “reading self-evaluation” scale reveal a noticeable asymmetry toward the agreement side of the scale, the distribution of “reading enjoyment” appears to be rather symmetrical. Since erroneous parameter estimates nevertheless occurred for both scales, this application example highlights that visual inspections of observed item responses are not necessarily indicative of mimicry effects, and should not be used as the sole diagnostic criterion for choosing the IRTree model applied to the data.

In an additional analysis, both scales were modeled simultaneously, which is generally preferable to the separate analysis of multiple scales. Since a joint model, which defines a response style as a category preference across several unrelated constructs, facilitates separating response style and trait factors more accurately (e.g., Bolt & Newton, 2011), this approach should also reduce mimicry effects. For the PISA data, the mimicry effects were indeed reduced in the joint model: The difference in the estimated correlation between trait and ERS by the IRTree model with unidimensional versus multidimensional pseudo-items decreased from .22 to .19 for “reading self-evaluation” and from .11 to .08. for “reading enjoyment.” The fact that mimicry effects were still present and of substantial size, however, is likely due to the high correlation of the two content traits of .48. Therefore, also when jointly analyzing multiple scales, IRTree models with multidimensional pseudo-items should be considered.

## Discussion

This article investigated the separation of substantive traits and response styles in IRTree models and addressed the threat of mimicry effects, a methodological artifact where response styles mimic the trait and capture trait-induced variance in item responding. As the response style factor functions as a substitute for the trait in such instances, the meaning of the estimated response styles does not correspond to the meaning that was assigned to the parameters when defining the model. Mimicry effects are manifested in a biased estimation of the covariance between response style and trait, with the bias being stronger the more the meanings of the two factors overlap. The covariance can be overestimated as well as underestimated, both of which can lead to severely misleading conclusions about the relationship between personal characteristics. For example, the IRTree model estimates may suggest that high levels of the trait of interest are associated with preferences for specific categories, although there is no or even an opposite relationship between these parameters. In addition to the biased estimation of the covariance, mimicry effects were found to be accompanied by inflated estimates of response style variances, meaning that the impact of a response style on the response selection is overestimated. In extreme cases, IRTree models might even misjudge the dimensionality of the data-generating process and indicate an influence of response styles where respondents actually provided purely trait-based responses. It could thus be concluded that some respondents did not work on the questionnaire with full effort but relied heavily on their response styles, although they engaged in an optimal and desired way of response selection. Particularly when dealing with high-stakes data such as assessments in job interviews, the false assumption that some applicants have made little effort to complete the task comes with potentially negative implications for such individuals and jeopardizes fairness. Consequently, it is important for both research and practice to be aware of possible methodological artifacts in IRTree models and to question the assigned meaning of estimated parameters rather than to interpret them as substantially meaningful without further consideration.

### Conditions and Implications of Mimicry Effects

An important research question is, therefore, under which conditions IRTree models pose the risk of artificial estimates. Our investigations suggested that only those IRTree models evoke mimicry effects, which are misspecified in a way that they overstate the influence of response styles and understate the influence of the trait in some pseudo-items. For example, this is the case if the item responses of a given data set originated from a unidimensional trait-based process, though an IRTree model with trait and ERS influences is applied. In such cases, the ERS factor can be used as a substitute for the trait and explain the trait-induced variance in extreme responding; in other words, a mimicry effect arises. In addition, the simulation studies corroborated our hypothesis that mimicry effects are largely dependent on the distribution of ordinal item responses across the rating scale. If they are symmetrically distributed with similar frequencies of agreement and disagreement categories, unbiased estimates of the variance-covariance matrix are provided. As such symmetrical response patterns yield no statistical advantage of a redeclaration of the response style parameter as a substitute for the trait, mimicry effects do not occur. In contrast, the more asymmetrically the responses are distributed across the scale, the better the variance in extreme responding can be explained if the response style parameters mimic the trait and capture trait-induced variance. As a result, mimicry effects occur and the meaningful separation of trait and response style parameters is compromised.

In the simulation studies, we operationalized the asymmetry of item responses by generating distributions of the trait levels which deviated from those of the item locations, through specifying either shifted mean structures or skewed distributions. Both led to considerable mimicry effects and an overestimation of the impact of response styles, even for small deviations between the distributions. This finding is highly relevant, as it is certainly not uncommon to apply a questionnaire to a group of individuals for whom it can be assumed that their traits are at least slightly differently distributed from that of the items. The empirical application to PISA data supported the results of the simulations, as indeed, even a small asymmetry of the observed responses was found to result in a mimicry effect.

Besides the threat of biased interpretations when analyzing the data of a single group of respondents, mimicry effects may likewise distort comparisons between multiple groups if such differ in their trait distributions. An example is cross-national assessments, for which one would certainly expect group differences in the distributions of the measured constructs, which would cause also the size of possible mimicry effects to vary. Though the comparisons of the trait of interest would not be impaired by mimicry effects, one may conclude that the groups differed in the extent of using response style (e.g., supposedly caused by different cultural backgrounds and socializations). IRTree models should thus be used with caution when shifted or skewed trait distributions may be present in the data, which is likely the case for many applications across all fields of psychology in which self-reported data are analyzed (e.g., clinical, personality, or work psychology).

However, this article also showed that not all types of IRTree models were prone to mimicry effects. The concerns and criticisms outlined above referred to the commonly used IRTree models in which all pseudo-items are parameterized by unidimensional models. Such models are based on the assumption that each sub-decision is affected by only one personal characteristic, which can be the trait or a response style. A different assumption is underlying IRTree models with multidimensional pseudo-items, in which the sub-decisions can be assigned several person parameters (e.g., the trait plus a response style). The simulation studies demonstrated that if the trait is additionally included in a pseudo-item, in which a response style would mimic the trait in the standard IRTree model with unidimensional parameterization, the trait itself accounts for the trait-induced variance, and the mimicry effect is prevented. The ability of such IRTree models to counteract mimicry effects was apparent in all simulation conditions of generated trait distribution, that is, was independent of the symmetry or asymmetry of the response distribution.

Furthermore, the advantage of a multidimensional parameterization of pseudo-items was not only evident for unidimensional, trait-based data-generating processes but also for more realistic multidimensional ones. We generated data under a two-dimensional IRTree model, in which the extreme pseudo-items were influenced by the ERS and the trait. Regardless of the true covariance of response style and trait, the IRTree model with multidimensional pseudo-items provided unbiased estimates and accurately reflected their true relationship. In contrast, the standard IRTree model with unidimensional pseudo-items led to mimicry effects whenever the response distribution was asymmetrical, although the size of such mimicry effects for multidimensional data was smaller compared with the effects in unidimensional data. This comparatively smaller mimicry effect indicates that the response style parameters are used to capture variance of both trait-based and response style-based responding for multidimensional data, and therefore, have less overlap with the trait compared with unidimensional data. Even though the potential for misinterpretations was consequently less severe under the more realistic multidimensional data, a disadvantage of unidimensional pseudo-items compared with multidimensional ones was still evident.

Despite improved psychometric properties of the models with multidimensional pseudo-items, the simulation studies also showed that the trait recovery was hardly affected by mimicry effects and biased response style estimates. Accordingly, the main purpose of response style modeling in empirical research and practice, namely, to obtain unbiased trait measurements, was successfully realized by both unidimensional and multidimensional parameterizations. Nevertheless, applying a model that yields biased estimates under certain circumstances of misspecification, even if such parameters are not of interest, should generally be avoided, as such a model is unable to provide information on the true data-generating process.

### Recommendations for the Specification of IRTree Models

Therefore, this article provides some suggestions on how to specify IRTree models and how to adapt them to the given research question and data: First, knowledge about the construct to be measured and about the questionnaire that is applied helps to anticipate whether the distributions of items and traits are likely to match or deviate from each other. Such theoretical considerations give an indication of whether using a standard IRTree model with unidimensional pseudo-items carries a risk of mimicry effects even before the data are collected. After data collection, it should be examined whether the empirical distribution of the item responses is symmetrical or asymmetrical. However, the application example demonstrated that even a slight asymmetry of responses, which can be easily overlooked or considered negligible, can lead to mimicry effects and change the interpretation of results. Unexpectedly high correlations of traits and response styles could thus be regarded as a warning sign for a possible mimicry effect. Nonetheless, mimicry effects can likewise result in an artificial reduction of an estimated relationship, which is probably a less obvious warning sign. We therefore recommend that IRTree models with unidimensional pseudo-items should only be applied if the trait distribution matches that of the items well, or if the response style estimates and the relationships between person parameters are not of interest for answering the research question. Of course, there may be certain hypotheses to be tested that require the specification of unidimensional processes, or only purely unidimensional sub-decisions are reasonable from a theoretical point of view. In such cases, it could be advisable to define an IRTree model across several questionnaire scales, though the benefits may be limited if the traits are correlated, as was evident in the application example. Therefore, further investigations may be needed to clarify how and to what extent the occurrence of mimicry effects can be reduced by simultaneously modeling several traits.

As a result, our analyses indicate that a multidimensional parameterization of pseudo-items should be generally preferred to a unidimensional one whenever possible. The advantage of multidimensional pseudo-items is all the more apparent since a possible overparameterization (e.g., using a two-dimensional parameterization for unidimensional pseudo-items) has no negative effect on the parameter estimation, as a non-existent influence of one of the person parameters is successfully detected by IRTree models. Moreover, the sub-decisions may actually be the result of a multidimensional response process, in which case only multidimensional pseudo-items can correctly reflect the true data-generating process. We thus believe that the prevention of mimicry effects and the greater flexibility of multidimensional parameterizations of pseudo-items outweigh the slightly higher modeling complexity in comparison to unidimensional pseudo-items. Furthermore, multidimensional pseudo-items can be readily implemented in standard software with little additional effort (like in the R package *mirt*; see the Online Supplementary Materials for *mirt* code of various IRTree models).

### Outlook

One limitation of this work is that we only considered one response style, the ERS, which is one of the most studied response styles in the literature. However, mimicry effects are probably also relevant for modeling other types of response styles such as the MRS. A classical IRTree model including MRS-based judgments defines three sub-decisions for responding to 6-point items, which are the decisions of agreement, moderate responding, and extreme responding (e.g., Böckenholt, 2017; Meiser et al., 2019): Respondents are assumed to first decide on whether they agree or disagree with the item and subsequently make an MRS-based sub-decision for midscale versus non-midscale responding conditional on agreement. In case they chose the non-midscale option, they decide on the extremity of their response based on their ERS. Just as derived before for the ERS, the meaning of the MRS is separated from that of the trait by defining a unique influence of the MRS across two pseudo-items (conditional on agreement and disagreement). Shifted or skewed trait distributions and asymmetrical response distributions should therefore most likely impair the separation of trait and MRS parameters and lead to mimicry effects also for the MRS. Still, the midscale pseudo-items can likewise be parameterized by multidimensional IRT models including an additional trait influence, which should counteract mimicry effects as successfully as shown here for the ERS. Although mimicry effects are thus likely to generalize to other response styles, it nevertheless remains to be clarified how they affect the parameter estimation of IRTree models when several response styles (e.g., ERS and MRS) are jointly modeled.

Furthermore, this article investigated mimicry effects only in IRTree models with a symmetrical tree structure (also called nested IRTree models), in which the same sequence of response processes is assumed to underlie the selection of corresponding categories on both sides of the rating scale. However, IRTree models can also be defined to have an asymmetrical structure (for an overview of different kinds of IRTree models, see Jeon & De Boeck, 2016). An example of such an asymmetrical IRTree model is the commonly used decomposition of 5-point rating items, in which one sub-decision represents the MRS-based choice to select either the neutral middle response category or one of the other categories. Conditional on the selection of a clear-cut category, two sub-decisions of trait-based agreement and ERS-based extreme responding are specified (Böckenholt, 2012; also see Khorramdel & von Davier, 2014; Plieninger, 2020; Zettler et al., 2016). In contrast to the models used in this article, the MRS is separated from the trait by means of only one pseudo-item. Such a model structure can be expected to likewise lead to mimicry effects for asymmetrical response distributions, though this should be systematically investigated and quantified and future work.

Various other modeling choices have been made in this article, the findings of which can be straightforwardly generalized to other choices: First, all IRTree models considered here were parameterized by the Rasch model, though other IRT models such as the 2PL model should naturally lead to similar results regarding mimicry effects. Furthermore, challenges in the separation of person parameters can be expected to not only occur for shifted or skewed trait distributions but also if traits and items mismatch otherwise. An example is bimodal trait distributions, which could result from an unknown mixture of two populations. Finally, mimicry effects can even be generalized beyond the IRTree model class to multidimensional ordinal IRT models such as the multidimensional nominal response model or the multidimensional PCM (e.g., Bolt et al., 2014; Falk & Cai, 2016 for an overview, see Henninger & Meiser, 2020). Just as for IRTree models, the meaningful separation of traits and response styles in such models is facilitated through uniquely directed influences of the person parameters, which can be assumed to be impaired if the distributions of the trait levels and item locations deviate from each other.

Overall, this article presented compelling evidence for the risk of mimicry effects in commonly used IRTree models. To address these concerns, we made suggestions on how to detect the lack of meaningful separation of traits and response styles and showed that IRTree models with multidimensional pseudo-items effectively counteract such mimicry effects. Our findings highlight the importance of being aware of potential methodological artifacts when modeling item response data and underline that further research is needed to ensure the validity of conclusions drawn from such data.

## Supplemental Material

sj-pdf-1-epm-10.1177_00131644231213319 – Supplemental material for Separation of Traits and Extreme Response Style in IRTree Models: The Role of Mimicry Effects for the Meaningful Interpretation of Estimates Supplemental material, sj-pdf-1-epm-10.1177_00131644231213319 for Separation of Traits and Extreme Response Style in IRTree Models: The Role of Mimicry Effects for the Meaningful Interpretation of Estimates by Viola Merhof, Caroline M. Böhm and Thorsten Meiser in Educational and Psychological Measurement

## Notes

1. We also compared the recovery of item difficulty parameters, although this is of limited relevance for most practical applications. The results of the item difficulty recovery for all simulation studies can be found in the Online Supplementary Materials.

2. The R code for generating data sets can be found on OSF: https://osf.io/9raud/.

3. The *mirt* code for such models can be found in the Online Supplementary Materials.

4. The data are openly available here: https://www.oecd.org/pisa/data/2018database/. The background questionnaire with all items can be found here: https://nces.ed.gov/surveys/pisa/pisa2018/questionnaires/Student_Q_Booklet_English.html.
