# Supplementary material for: Separation of Traits and Extreme Response Style in IRTree Models: The Role of Mimicry Effects for the Meaningful Interpretation of Estimates
Source: Educ Psychol Meas. 2023 Dec 22;84(5):927–56. doi: 10.1177/00131644231213319 (PMC11418598; doi:10.1177/00131644231213319)
Supplement: sj-pdf-1-epm-10.1177_00131644231213319 – Supplemental material for Separation of Traits and Extreme Response Style in IRTree Models: The Role of Mimicry Effects for the Meaningful Interpretation of Estimates [file sj-pdf-1-epm-10.1177_00131644231213319.pdf]

Supplementary Material A:

Additional Material Concerning the Simulation Studies

Table A1

Mean Estimated Covariance of ERS η and Trait θ by the IRTree Model for Unidimensional Data [Simulation 1]

Trait distribution		N=100				N=500				N=2000			
Distr. fam.	Condition	I=5	I=10	I=20	I=40	I=5	I=10	I=20	I=40	I=5	I=10	I=20	I=40
Shifted	$\mu = 0.0$	-0.06	0.01	-0.01	0.04	0.03	-0.02	-0.02	0.01	-0.01	0.00	0.01	0.00
	$\mu = 0.2$	0.13	0.20	0.15	0.14	0.16	0.15	0.16	0.14	0.17	0.10	0.17	0.12
	$\mu = 0.5$	0.27	0.43	0.37	0.37	0.34	0.34	0.35	0.33	0.38	0.36	0.35	0.35
	$\mu = 1.0$	0.70	0.66	0.72	0.73	0.66	0.69	0.69	0.71	0.74	0.69	0.70	0.70
Skewed	$\alpha = 0.0$	-0.01	-0.02	0.01	0.00	0.07	0.02	0.01	0.01	0.06	-0.02	0.01	0.01
	$\alpha = 0.5$	0.30	0.27	0.32	0.27	0.35	0.29	0.26	0.30	0.23	0.28	0.26	0.28
	$\alpha = 1.0$	0.57	0.59	0.56	0.58	0.52	0.50	0.54	0.53	0.52	0.53	0.55	0.54
	$\alpha = 2.0$	0.78	0.89	0.87	0.91	0.78	0.83	0.86	0.86	0.79	0.81	0.84	0.86

Table A2

Mean Trait Recovery $Cor(\theta, \hat{\theta})$ for Unidimensional Data [Simulation 1]

Analysis	Trait distribution		N=100				N=500				N=2000			
	Distr. family	Condition	I=5	I=10	I=20	I=40	I=5	I=10	I=20	I=40	I=5	I=10	I=20	I=40
IRTree	Shifted	$\mu = 0.0$	.76	.86	.92	.96	.76	.86	.92	.96	.77	.86	.92	.96
		$\mu = 0.2$	.76	.85	.92	.96	.77	.86	.92	.96	.77	.86	.92	.96
		$\mu = 0.5$	.76	.86	.92	.96	.77	.86	.92	.96	.77	.86	.92	.96
		$\mu = 1.0$	.77	.86	.92	.95	.77	.86	.92	.95	.77	.86	.92	.95
	Skewed	$\alpha = 0.0$	.77	.86	.92	.96	.77	.86	.92	.96	.76	.86	.92	.96
		$\alpha = 0.5$	.77	.85	.92	.96	.77	.86	.92	.96	.77	.86	.92	.96
		$\alpha = 1.0$	.76	.86	.92	.96	.77	.86	.92	.96	.77	.86	.92	.96
		$\alpha = 2.0$	.76	.86	.92	.96	.76	.86	.92	.96	.77	.86	.92	.96
PCM	Shifted	$\mu = 0.0$	.82	.90	.95	.97	.83	.90	.95	.97	.83	.90	.95	.97
		$\mu = 0.2$	.83	.90	.95	.97	.83	.90	.95	.97	.83	.90	.95	.97
		$\mu = 0.5$	.83	.90	.95	.97	.83	.90	.95	.97	.83	.90	.95	.97
		$\mu = 1.0$	.82	.90	.94	.97	.82	.90	.94	.97	.82	.90	.94	.97
	Skewed	$\alpha = 0.0$	.83	.90	.95	.97	.83	.91	.95	.97	.83	.90	.95	.97
		$\alpha = 0.5$	.83	.90	.95	.97	.83	.90	.95	.97	.83	.90	.95	.97
		$\alpha = 1.0$	.82	.90	.95	.97	.83	.90	.95	.97	.83	.90	.95	.97
		$\alpha = 2.0$	.81	.89	.94	.97	.81	.89	.94	.97	.81	.89	.94	.97

Table A3

Mean Estimated Covariance of ERS η and Trait θ by the IRTree Model for Multidimensional Data with ERS Influence [Simulation 3]

Cov(η, θ)	Condition	N=100				N=500				N=2000			
		I=5	I=10	I=20	I=40	I=5	I=10	I=20	I=40	I=5	I=10	I=20	I=40
0.0	$\mu = 0.0$	-0.02	0.02	0.02	0.01	0.01	0.00	0.00	0.00	-0.01	-0.02	-0.00	-0.00
	$\mu = 1.0$	0.12	0.16	0.14	0.16	0.16	0.14	0.14	0.15	0.13	0.15	0.15	0.14
0.2	$\mu = 0.0$	0.20	0.20	0.20	0.21	0.18	0.19	0.18	0.19	0.21	0.19	0.20	0.19
	$\mu = 1.0$	0.30	0.34	0.37	0.35	0.34	0.33	0.32	0.34	0.32	0.34	0.32	0.34
0.4	$\mu = 0.0$	0.42	0.40	0.38	0.37	0.40	0.37	0.37	0.39	0.37	0.36	0.37	0.39
	$\mu = 1.0$	0.60	0.57	0.53	0.51	0.53	0.52	0.55	0.54	0.53	0.54	0.52	0.53
0.6	$\mu = 0.0$	0.56	0.57	0.59	0.56	0.59	0.55	0.57	0.58	0.58	0.59	0.56	0.56
	$\mu = 1.0$	0.76	0.70	0.78	0.74	0.74	0.73	0.71	0.73	0.70	0.72	0.72	0.71

Table A4

Mean Trait Recovery Cor(θ, $\hat{\theta}$) for Multidimensional Data [Simulation 3]

Analysis	Trait shift	N=100				N=500				N=2000			
		I=5	I=10	I=20	I=40	I=5	I=10	I=20	I=40	I=5	I=10	I=20	I=40
IRTree	$\mu = 0.0$	.65	.77	.86	.92	.66	.77	.86	.92	.66	.77	.86	.92
	$\mu = 1.0$	.65	.77	.86	.92	.66	.77	.86	.92	.66	.77	.86	.92
IRTree	$\mu = 0.0$	.64	.79	.88	.94	.69	.80	.88	.94	.69	.80	.88	.94
multidim.	$\mu = 1.0$	.64	.78	.88	.93	.68	.80	.88	.93	.69	.80	.88	.93
PCM	$\mu = 0.0$	.67	.78	.87	.92	.67	.78	.87	.93	.67	.79	.87	.93
	$\mu = 1.0$	.67	.78	.87	.92	.67	.79	.87	.92	.68	.79	.87	.92

Table A5

Mean ERS Recovery Cor(η, $\hat{\eta}$) for Multidimensional Data [Simulation 3]

Analysis	Trait shift	N=100				N=500				N=2000			
		I=5	I=10	I=20	I=40	I=5	I=10	I=20	I=40	I=5	I=10	I=20	I=40
IRTree	$\mu = 0.0$	.63	.75	.85	.91	.64	.76	.85	.91	.64	.76	.85	.91
	$\mu = 1.0$	.63	.75	.85	.90	.64	.76	.85	.90	.64	.76	.85	.90
IRTree	$\mu = 0.0$	.57	.75	.86	.92	.65	.77	.86	.92	.65	.77	.86	.92
multidim.	$\mu = 1.0$	.58	.75	.86	.92	.65	.77	.86	.92	.65	.77	.86	.92

Supplementary Material B:
Recovery of Item Difficulty Parameters

Table B1

Recovery of Item Difficulties $Cor(\beta, \hat{\beta})$ for Unidimensional and Multidimensional Data

Data	Cov(η, θ)	Trait distribution		*M* (*SD*) across replications		
		Distr. family	Condition	PCM	IRTree	IRTree multidim.
Unidim.	–	Shifted	$\mu = 0.0$	.99 (.01)	.92 (.04)	.98 (.01)
(Sim. 1 & 2)			$\mu = 1.0$	.98 (.02)	.84 (.07)	.97 (.02)
	–	Skewed	$\alpha = 0.0$	.99 (.01)	.92 (.04)	.98 (.01)
			$\alpha = 2.0$	.98 (.02)	.85 (.07)	.97 (.02)
Multidim.	0.0	Shifted	$\mu = 0.0$	.91 (.03)	.98 (.02)	.99 (.02)
(Sim. 3)			$\mu = 1.0$	.91 (.03)	.96 (.02)	.97 (.02)
	0.2	Shifted	$\mu = 0.0$	.91 (.03)	.98 (.02)	.99 (.02)
			$\mu = 1.0$	.91 (.03)	.96 (.02)	.97 (.02)
	0.4	Shifted	$\mu = 0.0$	.91 (.03)	.98 (.02)	.99 (.02)
			$\mu = 1.0$	.91 (.03)	.96 (.02)	.97 (.02)
	0.6	Shifted	$\mu = 0.0$	.91 (.03)	.98 (.02)	.99 (.02)
			$\mu = 1.0$	.91 (.03)	.96 (.02)	.97 (.02)

Note. $N = 500$, $I = 20$. The evaluated item parameters were the item- and category-specific difficulties under the PCM (β_{ik} in Equation 2) and the pseudo-item difficulties under the IRTree model (β_{ih} in Equation 1). Due to the different ways of coding item responses in the PCM and IRTree model, the associated pairs of item difficulties are the following: (PCM: β_{i1}/ IRTree: β_{i3}); (PCM: β_{i2}/ IRTree: β_{i1}); (PCM: β_{i3}/ IRTree: β_{i2}).

Supplementary Material C:
Additional Simulation Study on the Asymmetry of the Response Distribution

Table C1

Estimated Covariances and Correlations of ERS η and Trait θ by the IRTree Model for Response Distributions with an Asymmetry Toward High and Low Categories

Trait distribution		M (SD) across replications			
Distr. family	Condition	$\widehat{\mathrm{Cov}}(\eta, \theta)$		$\widehat{\mathrm{Cor}}(\eta, \theta)$	
		Asym. high (+)	Asym. low (–)	Asym. high (+)	Asym. low (–)
Shifted	$\mu = 0.0$	0.00 (0.26)	0.00 (0.26)	0.00 (0.34)	-0.01 (0.34)
	$\mu = \pm 0.2$	0.15 (0.26)	-0.15 (0.26)	0.20 (0.34)	-0.20 (0.32)
	$\mu = \pm 0.5$	0.35 (0.27)	-0.36 (0.27)	0.44 (0.29)	-0.44 (0.29)
	$\mu = \pm 1.0$	0.70 (0.26)	-0.71 (0.24)	0.74 (0.18)	-0.74 (0.18)
Skewed	$\alpha = 0.0$	0.01 (0.26)	-0.01 (0.29)	0.01 (0.34)	-0.01 (0.35)
	$\alpha = \pm 0.5$	0.28 (0.26)	-0.29 (0.27)	0.36 (0.30)	-0.36 (0.31)
	$\alpha = \pm 1.0$	0.54 (0.27)	-0.55 (0.25)	0.62 (0.25)	-0.63 (0.22)
	$\alpha = \pm 2.0$	0.84 (0.23)	-0.82 (0.24)	0.83 (0.13)	-0.83 (0.14)

Note. Aggregated across sample sizes (N = 100, 500, 2000) and questionnaire lengths (I = 5, 10, 20, 40).

Supplementary Material D:
Model Estimation in *mirt*

Listing 1

Set Up mirt Package and Load Data

```
install.packages("mirt")                    # install mirt package
library(mirt)                               # load mirt package
load("example_data.RDA")                    # load data
I <- example_data$I                         # number of items I=20
condition <- example_data$condition         # trait shift = 0.5

head(example_data$ordinal_responses) # ordinal responses
## V1  V2  V3  V4  V5  V6  V7  V8  V9  V10 V11 V12 V13 V14 V15 V16 V17 V18 V19 V20
## 2   2   3   3   2   3   2   1   3   2   2   1   2   1   3   2   1   1   2   3
## 1   2   3   3   1   3   3   1   1   3   1   3   2   0   2   1   2   1   2   3
## 3   3   3   2   2   2   3   1   3   2   1   3   3   2   2   0   1   2   1   2
## 1   1   3   2   1   2   3   1   3   2   0   1   2   1   0   0   1   1   1   0
## 1   3   3   3   2   3   3   2   2   3   1   0   3   1   2   1   1   1   1   1
## 1   2   3   2   2   3   2   3   2   2   0   2   2   1   3   2   1   3   1   1

head(example_data$pseudo_item_responses) # pseudo-item responses
## V1  V2  V3  V4  V5  V6  V7  V8  V9  V10 V11 V12 V13 V14 V15 V16 V17 V18 V19 V20
## NA  NA  NA  NA  NA  NA  NA  0   NA  NA  NA  0   NA  0   NA  NA  0   0   NA  NA
## 0   NA  NA  NA  0   NA  NA  0   0   NA  0   NA  NA  1   NA  0   NA  0   NA  NA
## NA  NA  NA  NA  NA  NA  NA  0   NA  NA  0   NA  NA  NA  NA  1   0   NA  0   NA
## 0   0   NA  NA  0   NA  NA  0   NA  NA  1   0   NA  0   1   1   0   0   0   1
## 0   NA  NA  NA  NA  NA  NA  NA  NA  NA  0   1   NA  0   NA  0   0   0   0   0
## 0   NA  NA  NA  NA  NA  NA  NA  NA  NA  1   NA  NA  0   NA  NA  0   NA  0   0
## V21 V22 V23 V24 V25 V26 V27 V28 V29 V30 V31 V32 V33 V34 V35 V36 V37 V38 V39 V40
## 1   1   1   1   1   1   1   0   1   1   1   0   1   0   1   1   0   0   1   1
## 0   1   1   1   0   1   1   0   0   1   0   1   1   0   1   0   1   0   1   1
## 1   1   1   1   1   1   1   0   1   1   0   1   1   1   1   0   0   1   0   1
## 0   0   1   1   0   1   1   0   1   1   0   0   1   0   0   0   0   0   0   0
## 0   1   1   1   1   1   1   1   1   1   0   0   1   0   1   0   0   0   0   0
```

```
## 0    1    1    1    1    1    1    1    1    1    0    1    1    0    1    1    0    1    0    0
## V41  V42  V43  V44  V45  V46  V47  V48  V49  V50  V51  V52  V53  V54  V55  V56  V57  V58  V59  V60
## 0    0    1    1    0    1    0    NA   1    0    0    NA   0    NA   1    0    NA   NA   0    1
## NA   0    1    1    NA   1    1    NA   NA   1    NA   1    0    NA   0    NA   0    NA   0    1
## 1    1    1    0    0    0    1    NA   1    0    NA   1    1    0    0    NA   NA   0    NA   0
## NA   NA   1    0    NA   0    1    NA   1    0    NA   NA   0    NA   NA   NA   NA   NA   NA   NA
## NA   1    1    1    0    1    1    0    0    1    NA   NA   1    NA   0    NA   NA   NA   NA   NA
## NA   0    1    0    0    1    0    1    0    0    NA   0    0    NA   1    0    NA   1    NA   NA

# V1–V20:
# extreme pseudo–item conditional on disagreement (ordinal categories 0 vs. 1)
# category 0 is coded with 1 (extreme response)
# category 1 is coded with 0 (non–extreme response)

# V21–V40:
# agreement pseudo–item (ordinal categories 0/1 vs. 2/3)
# categories 0/1 are coded with 0 (disagreement)
# categories 2/3 are coded with 1 (agreement)

# V41–V60:
# extreme pseudo–item conditional on agreement (ordinal categories 2 vs. 3)
# category 2 is coded with 0 (non–extreme response)
# category 3 is coded with 1 (extreme response)
```

Listing 2

Standard IRTree Model with Unidimensional Pseudo-Items

```
model_IRTree <- mirt.model(paste0("theta=", I+1, "-", 2*I ,"\n",
                                  "eta=", 1, "-", I, ",", 2*I+1, "-", 3*I, "\n",
                                  "COV = theta*eta"))
model_IRTree
## $x
##      Type    Parameters
## [1,] "theta" "21-40"
## [2,] "eta"   "1-20,41-60"
## [3,] "COV"   "theta*eta"
##
## attr(,"class")
## [1] "mirt.model"

fit_IRTree <- mirt(data=example_data$pseudo_item_responses, model=model_IRTree,
                   itemtype="Rasch", method = "EM")
# For the standard IRTree model, we found that the default EM estimation method
# works fine for most of the models. In case the model does not converge, the
# quasi-Monte Carlo EM estimation (method = "QMCEM") might help.

fit_IRTree # Check whether the model converged.
## Full-information item factor analysis with 2 factor(s).
## Converged within 1e-04 tolerance after 58 EM iterations.
## mirt version: 1.38.1
## M-step optimizer: nlminb
## EM acceleration: Ramsay
## Number of rectangular quadrature: 31
## Latent density type: Gaussian
##
## Log-likelihood = -9980.221
## Estimated parameters: 63
## AIC = 20086.44
## BIC = 20351.96; SABIC = 20152
```

```
coef(fit_IRTree, simplify=T)
## $items
##        a1  a2       d  g  u
## V1      0   1  -0.504  0  1
## V2      0   1  -1.143  0  1
## V3      0   1  -1.324  0  1
## [...]
## V20     0   1  -0.995  0  1
## V21     1   0   0.035  0  1
## V22     1   0   1.483  0  1
## V23     1   0   3.485  0  1
## [...]
## V40     1   0   1.273  0  1
## V41     0   1  -0.197  0  1
## V42     0   1   0.185  0  1
## V43     0   1   1.383  0  1
## [...]
## V60     0   1  -0.268  0  1

## $means
## theta   eta
##     0     0

## $cov
##        theta    eta
## theta  2.054  0.535
## eta    0.535  0.253

# Note: mirt uses the slope-intercept form, not the traditional IRT parameters:
# a1 = slope of theta (trait)
# a2 = slope of eta (ERS)
# d = intercept
# For the IRTree model, the item difficulty b is equal to -d.
```

Listing 3

IRTree Model with Fixed Covariance and Unidimensional Pseudo-Items

```
model_IRTree_fixed_cov <- mirt.model(paste0("theta=", I+1, "-", 2*I ,"\n",
                                            "eta=", 1, "-", I, ",", 2*I+1, "-", 3*I))
model_IRTree_fixed_cov
## $x
##        Type     Parameters
## [1,] "theta" "21-40"
## [2,] "eta"   "1-20,41-60"
##
## attr(,"class")
## [1] "mirt.model"

fit_IRTree_fixed_cov <- mirt(data=example_data$pseudo_item_responses,
                             model=model_IRTree_fixed_cov,
                             itemtype="Rasch", method = "EM")
fit_IRTree_fixed_cov
## [...]
## Converged within 1e-04 tolerance after 33 EM iterations.
## [...]

coef(fit_IRTree_fixed_cov,  simplify=T)
## $items
##      a1 a2      d g u
## V1    0  1 -0.633 0 1
## V2    0  1 -1.346 0 1
## V3    0  1 -1.693 0 1
## [...]
## V20   0  1 -1.169 0 1
## V21   1  0  0.006 0 1
## V22   1  0  1.459 0 1
## V23   1  0  3.498 0 1
##  [...]
## V40   1  0  1.247 0 1
## V41   0  1 -0.079 0 1
```

```
## V42   0   1    0.244  0  1
## V43   0   1    1.400  0  1
## [...]
## V60   0   1   -0.207  0  1

## $means
## theta    eta
##      0      0

## $cov
##        theta    eta
## theta  1.968  0.000
## eta    0.000  0.236
```

Listing 4

IRTree Model with Multidimensional Extreme Pseudo-Items

```
# recode first pseudo-item (extreme responding conditional on disagreement) in
# direction of trait:
# category 0 is coded with 0 (extreme response)
# category 1 is coded with 1 (non-extreme response)
example_data$pseudo_item_responses_recoded <- example_data$pseudo_item_responses
example_data$pseudo_item_responses_recoded[,1:I] <-
                                          1-example_data$pseudo_item_responses[,1:I]

model_IRTree_multi <- mirt.model(paste0(
                  "theta = 1-", 3*I, "\n",
                   "eta = ", 1, "-", I ,",", 2*I+1, "-", 3*I, "\n",
                   "START = (1-", I, ", a2, -1.0), (", I+1," -", 2*I, ", a1, 1.0),
                            (",2*I+1, "-" ,3*I, ", a2, 1.0) \n",
                   "FIXED = (1-" ,I, ", a2), (", (I+1)," -", 2*I, ", a1),
                            (",2*I+1, "-" ,3*I, ", a2) \n",
                   "CONSTRAIN = (", 1, "-" ,I, ",", 2*I+1," -", 3*I,", a1) \n",
                   "COV = theta*theta, eta*eta, theta*eta"
                   ))
model_IRTree_multi
## $x
##      Type        Parameters
## [1,] "theta"     "1-60"
## [2,] "eta"       "1-20,41-60"
## [3,] "START"     "(1-20,a2,-1.0),(21-40,a1,1.0),(41-60,a2,1.0)"
## [4,] "FIXED"     "(1-20,a2),(21-40,a1),(41-60,a2)"
## [5,] "CONSTRAIN" "(1-20,41-60,a1)"
## [6,] "COV"       "theta*theta,eta*eta,theta*eta"
##
## attr(,"class")
## [1] "mirt.model"

# Parameters can be fixed by the mirt commands "START" and "FIXED".
# As a 2PL model is fitted to estimate the discrimination parameter of the trait
```

```
# influence on extreme responding, the discrimination parameters of the other
# processes are manually set to 1 as given by the Rasch model.
# The discrimination parameter of eta for extreme responding conditional on
# disagreement is set to -1 to account for the recoded pseudo-item.
# The trait influence on extreme responding is set equal for the two extreme
# pseudo-items by the mirt command "CONSTRAIN".

fit_IRTree_multi <- mirt(data=example_data$pseudo_item_responses_recoded,
                         model=model_IRTree_multi,
                         itemtype="2PL",method = "MHRM")
# We found that the Metropolis-Hastings Robbins-Monro (MHRM) performed best
# for the IRTree model with multidimensional pseudo-items.
# The default EM estimation method was less effective.

fit_IRTree_multi
## [...]
## Converged within 0.001 tolerance after 49 MHRM iterations.
## [...]

coef(fit_IRTree_multi,  simplify=T)
## $items
##        a1 a2      d g u
## V1  0.694 -1  1.398 0 1
## V2  0.694 -1  2.326 0 1
## V3  0.694 -1  3.117 0 1
## [...]
## V20 0.694 -1  2.105 0 1
## V21 1.000  0  0.061 0 1
## V22 1.000  0  1.506 0 1
## V23 1.000  0  3.538 0 1
## [...]
## V40 1.000  0  1.295 0 1
## V41 0.694  1 -0.473 0 1
## V42 0.694  1  0.100 0 1
## V43 0.694  1  1.511 0 1
```

```
## [...]
## V60 0.694   1  -0.404 0 1

## $means
## theta     eta
##     0       0

## $cov
##          theta      eta
## theta   1.932  -0.044
## eta    -0.044   0.038
```

Listing 5

Unidimensional PCM

```
model_PCM <- mirt.model(paste0("theta = 1-", I))
model_PCM
## $x
##         Type          Parameters
## [1,] "theta"       "1-20"
##
## attr(,"class")
## [1] "mirt.model"

fit_PCM <- mirt(data=example_data$ordinal_responses,
                model=model_PCM, itemtype="Rasch")
fit_PCM
## [...]
## Converged within 1e-04 tolerance after 18 EM iterations.
## [...]

coef(fit_PCM, simplify=T)
## $items
##      a1 ak0 ak1 ak2 ak3 d0    d1    d2    d3
## V1    1   0   1   2   3  0 1.310 1.059 0.573
## V2    1   0   1   2   3  0 2.296 3.158 3.195
## [...]
## V19   1   0   1   2   3  0 3.697 3.688 1.341
## V20   1   0   1   2   3  0 2.072 2.937 2.467
##
## $means
## theta
##     0
##
## $cov
##       theta
## theta 0.921
```
